# Predictors of change in CD4 cell count over time for HIV/AIDS patients on ART follow-up in northern Ethiopia: a retrospective longitudinal study

**DOI:** 10.1186/s12865-024-00659-3

**Published:** 2024-10-04

**Authors:** Gebru Gebremeskel Gebrerufael, Zeytu Gashaw Asfaw

**Affiliations:** 1 Department of Statistics, College of Natural Science, Adigrat University, Adigrat, Ethiopia; 2 Department of Epidemiology and Biostatistics, School of Public Health, Addis Ababa University, Addis Ababa, Ethiopia

**Keywords:** HIV/AIDS, ART, CD4 cell count, Northern Ethiopia

## Abstract

**Background:**

HIV has an effect on lowering CD4 cell count, which lowers the ability to resist contamination. For patients on ART in areas with limited resources, the CD4 cell count assessment is crucial for determining treatment responses and therapeutic decisions. The volatility of CD4 counts following the introduction of ART over time is still largely uncharacterized, and there are few fresh datasets on CD4 cell count progressions. The goal of this study was to identify the key factors that change over time in CD4 cells for HIV/AIDS patients receiving ART follow-up in northern Ethiopia.

**Methods:**

A total of 216 HIV/AIDS patients who initiated ART in the Mekelle General Hospital between 2013 and 2016 were involved using systematic random selection techniques. An examination of exploratory data was used to describe the individual profiles of HIV patients. A multivariable random intercept and slope linear mixed regression analysis regarded predictor variables to be statistically significant if their *p*-value was less than 0.05.

**Results:**

The random intercept and slope linear mixed model result indicated that there were statistically significant predictors of baseline CD4 cell count (β = 0.0125, *P*-value = 0.001*) and bedridden functional status (β = -2.459, *p* = 0.02*) on the change of CD4 cell count over time in HIV/AIDS patients at the 5% significance level.

**Conclusions:**

Changes in CD4 counts were influenced by the baseline CD4 cell count and the functional status of being bedridden. Because their CD4 cell counts were lower at baseline and they had a functional status of bedridden, the majority of HIV/AIDS patients on ART had substantial predictors on the change of CD4 cell count over time.

So, public health service providers should give exceptional guidance and attention is also necessary for those patients who have lower baseline CD4 cell count and bedridden functional status.

**Supplementary Information:**

The online version contains supplementary material available at 10.1186/s12865-024-00659-3.

## Introduction

A significant global public health issue is Human Immunodeficiency Virus/Acquired Immune Deficiency Syndrome (HIV/AIDS) [1]. According to the 2023 UNAIDS report, there were 39.9 million people living with HIV/AIDS, 630,000 people died from infections related to HIV/AIDS, and 1.3 million people had the new HIV disease [2]. HIV has an effect on lowering CD4 level, which decreases ability to combat contamination [3].

One of the most severely affected regions of the world is Sub-Saharan Africa, where there are 22.9 million people living with HIV/AIDS and 1.2 million deaths from the disease among adults and teenagers in 2010. One of the Sub-Saharan African nations most severely impacted by HIV/AIDS is Ethiopia [4]. In Ethiopia, there were about 710,000 infected people, 404,405 HIV/AIDS patients were being monitored on antiretroviral therapy, and 20,000 patient deaths were reported in 2016 [5]. The country’s economic progress has slowed down as a result of the high death rate among HIV/AIDS sufferers [6].

According to estimates, 56,900 HIV/AIDS patients in the Tigray Regional State are still alive and had improved survival rates and quality of life in 2012 [7, 8].

Numerous researchers have examined variations in patients’ CD4 cells who are HIV positive. However, those researchers have observed differences in cross-sectional study points of view without observing any potential correlations between successively repeated changes in CD4 cells [9–14]. Both multiple regression model analysis and logistic regression, two popular regression models, were used in these investigations. Therefore, it is crucial to conduct research that uses a linear mixed model to examine the relationship between recurring CD4 cells and predictors [15]. Moreover, these researchers mainly considered repeatedly measured of variation CD4 cells for the same patient was independent of each other when determining predictors of CD4 Cells in HIV/AIDS patients receiving Antiretroviral Therapy (ART), CD4 Cells and Plasma HIV-1 positive of HAART, respectively [16]. Nevertheless, it was shown in this recent study using a longitudinal modeling technique that the pattern of advancement in repeated CD4 cell measurement for the same patient was dependent upon one another. Additionally, in settings with limited resources, the CD4 cell count measurement is crucial for determining how well patients are responding to treatment and for clinical decision-making. The volatility of CD4 counts following the introduction of ART over time is still largely uncharacterized, and there are few fresh datasets on CD4 cell count progressions [16]. Due to this, the goal of the current study was to pinpoint the main indicators of CD4 cell changes over time in HIV/AIDS patients who were being monitored. This model benefited from the effectiveness of parameter estimates. Therefore, optimizing such longitudinal model has methodological and practical contribution for health staff and used as reference for further studies indicates for its theoretical contribution. Identifying these predictors that affect change of CD4 cells on HIV/AIDS patients also helps health professionals to facilitate proper management and monitoring of the health care intervention on ART program.

## Methods

### Study design, data collection and study period

A retrospective longitudinal study conducted at an institution was used to gather relevant secondary data from the ART follow-up chart. In order to include at least two visit time responses after the beginning of their regiment, HIV/AIDS infected patients who are >15 years old and start ART between September 11, 2013 and September 5, 2016, CD4 cells measurement just before beginning of ART, were considered as predictors. Utilizing a standardized checklist, data were extracted. Using SPSS for data entry, coding, and cleaning, the secondary data were exported and analyzed in R version 3.6.1.

### Study area, population and sampling procedures

This information was gathered from HIV/AIDS patients who began taking ART between September 11, 2013, and September 5, 2016, at Mekelle General Hospital in Tigray Region State, Ethiopia. Mekelle, the state capital of the Tigray Region State, is located in northern Ethiopia and approximately 783 kilometers northwest of Addis Abeba. It also has an estimated 4,664,071 inhabitants overall, of which 2,367,032 are women. According to estimates, more over 80% of the population lives in rural areas. Tigray Region State has 14 hospitals and 205 health centers in 2012, according to a data from the regional health agency [7]. Out of 865 HIV/AIDS patients, a random sample of 216 was chosen utilizing systematic random selection methods.

### Variables of this study

#### Response variable

The key longitudinal outcome variable for this study was the change in CD4 cells in HIV-positive individuals after they started receiving ART. Patients’ CD4 cells were often noted in medical records.

#### Predictor variables

The sociodemographic and clinical ART factors that were recorded from the medical charts for this study are listed below on Table 1.

**Table 1:** Description, coding and categories of predictor variables

**Predictor variables**	**Categories and coding predictor variables**
Age in years	continuous
Sex	0 = female,1 = male
Baseline CD4 cell count	continuous
Baseline hemoglobin	continuous
Residence	0 = urban,1 = rural
WHO-clinical stage	0 = stage-I,1 = stage-II, 2 = stage-III, 3 = stage-IV
HIV/TB status	0 = no,1= yes
Functional status	0 = working,1 = fair, 2 = bedridden
Visiting time	discrete

### Statistical data analysis

One of the associated datasets is the examination of longitudinal data from CD4 cells. The measurements of the biomarkers are either constant or changing over time. All patients provided measurements at the same set of times, which were regularly and uniformly spaced, such as every six months in this study. While the times are altering, various treatments have been used for various individuals at various times [17–19]. In this study, the covariance structure and size of the residual errors were also examined for model selection, and the most preferred model was a random effect model with the least amount of individual variability.

Additionally, missing values are a frequent and difficult problem in longitudinal data analysis, and the most common imputation technique to manage missing values is multiple imputations.

The benefit of utilizing the linear mixed model (LMM) is that it makes use of all available data, including incomplete cases, and that it produces reliable and effective estimators of precision parameters. As a result, CD4 cell variability within and between patients was examined for each patient with i = 1, 2,... n [17, 20, 21].

1. Random intercept model

A model with random intercepts is one in which the intercepts are only allowed to vary and the change in the response variable for each subsequent measurement is anticipated.

1 $${\text{y}}_{ij}= {\beta }_{0} +{b}_{0i} + {\beta }_{1}{\text{t}}_{ij} + {\in }_{ij};$$

${b}_{0i}$~N (0,${{\delta }_{0}}^{2}$) , ${\in }_{ij}$~N (0,${\delta }^{2}$), ${b}_{0i}$ and ${\in }_{ij}$ are independent

2). Random slope model

In a model where slopes are allowed to vary, changes on the response variable for each subsequent measurement are anticipated by slope that varies between patients, the model is known as a random slope model.

2 $${\text{y}}_{ij}= {\beta }_{0} +{\beta }_{1}{t}_{ij} + {b}_{1i}{\text{t}}_{ij} + {\in }_{ij};$$

Where, ${b}_{1i}$~N (0,${{\delta }_{1}}^{2}$) ${\in }_{ij}$~N (0,${\delta }^{2}$) and ${b}_{1i}$ and ${\in }_{ij}$ are independent.

3). Random intercept and slope model

The most realistic kind of model is probably one with both random intercepts and random slopes (RI-RS). Both intercepts and slopes may differ between patient groups in this model [22].

3 $${\text{y}}_{ij}= {\beta }_{0} + {\beta }_{1}{\text{t}}_{ij} + {b}_{i1}{\text{t}}_{ij}+{b}_{i0}+ {\in }_{ij};$$

$$\left\{\begin{array}{l}\begin{array}{cc}{\text{b}}_{i}=\left[\begin{array}{c}{b}_{i0}\\ {b}_{i1}\end{array}\right]\sim N\left(0,{\Omega }_{b}\right)& (4\text{a})\end{array}\\ \begin{array}{cc}{\Omega }_{b}=\left[\begin{array}{c}{{\delta }_{{b}_{0}}}^{2} {\delta }_{b1} \\ {\delta }_{b1} {{\delta }_{{b}_{0}}}^{2} \end{array}\right]& (4\text{b})\end{array}\\ \begin{array}{l}\begin{array}{cc}{\in }_{i}\sim N\left(0,{{\delta }_{e}}^{2}\right)& (4\text{c})\end{array}\\ \begin{array}{cc}{\text{b}}_{i}\sim MVN\left(0,{\sum .}_{i}\right)& (4\text{d})\end{array}\\ \begin{array}{l}\begin{array}{ll}\text{V ar}\left({y}_{ij}\right)={{\delta }_{1}}^{2} + 2{\delta }_{01}{\text{t}}_{ij} + {{\delta }_{1}}^{2}{\text{t}}_{ij} +{\delta }^{2}& (4\text{e})\end{array}\\ \begin{array}{ll}Cov\left({y}_{ij},{y}_{ik}\right)={{\delta }_{0}}^{2} + {\delta }_{01}\left({\text{t}}_{ij},{\text{t}}_{ik}\right) + {{\delta }_{1}}^{2}{\text{t}}_{ij}, {\text{t}}_{ik} +{\delta }^{2} & (4\text{f})\end{array}\\ \begin{array}{c}\begin{array}{cc}\text{Cov}\left({y}_{ij},{y}_{lk}\right)= 0& (4\text{g})\end{array}\\ and\; {\text{b}}_{1},\dots ..,{\text{b}}_{n}, and\; {\in }_{1},\dots \dots .{\in }_{n}\; are\; independent\end{array}\end{array}\end{array}\end{array}\right.$$

### Choosing the best covariance structure and model selection criteria

The process of choosing a model from a group of candidate models is known as model selection. It is necessary to select a covariance model based on an anticipated mean response model. The researcher fitted models with straightforward structures, and it was preferred to choose models with the fewest AIC/BIC values in order to minimize the number of parameters in the variance-covariance structure. In this study, Independent (IND), Compound Symmetry (CS), Heterogeneous Compound Symmetry (CSH), First-Order Autoregressive (AR (1)), and Unstructured (UN) were the most often used. These models aid in making inferences about the mean parameters more effectively. The models with the minimum AIC/BIC were therefore the best fits [23–25].

## Results

### Descriptive characteristics of HIV/AIDS patients

The distribution of individual patients was shown by the descriptive analysis of the data using the various levels of predictors taken into account with their minimum and maximum CD4 cells (Table 2). A total of 216 HIV-positive patients were selected for the study, and 134 (62.04%) of those cases involved HIV-positive women. The majority of individuals (138 or 63.9%) had a pre-observation WHO clinical stage of III/IV and a functional status of “bedridden” (117 or 54.2%).

**Table 2:** Clinical and socio-demographic characteristic of independent variables

Categorical variables	Categories	Frequency (%)			
Sex	female	134(62.04%)			
	male	82(37.96%)			
Residence	urban	88(40.74%)			
	rural	128(59.25%)			
WHO clinical stage	stage-I	20(9.3%)			
	stage-II	58(26.9%)			
	stage-III	56(25.9%)			
	stage-IV	82(38%)			
HIV/TB status	no	167(77.3%)			
	yes	49(22.7%)			
Functional status	working	22(10.2%)			
	fair	77(35.7%)			
	bedridden	117(54.2%)			
**Continuous predictor variables**		**Minimum**	**Maximum**	**Median**	**SD**
Baseline CD4 cell count		50	982	314	161
Baseline age		17	68	32	10.9
Baseline hemoglobin		7.3	19.2	13.6	1.96
Visiting time		2	6		

### Exploring individual profile of patients

**Fig. 1:**
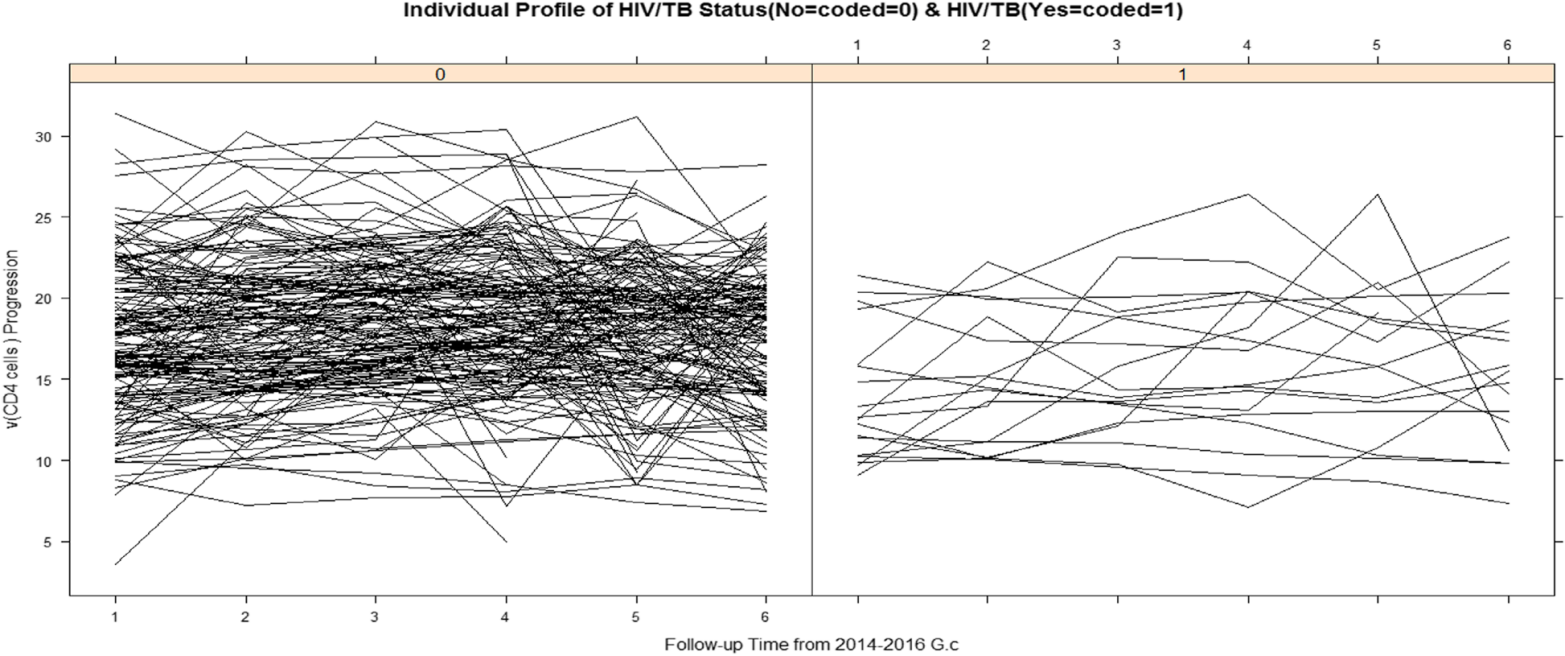
Shows individual profile separated by co-infection status (coded by no (-ve) =0, yes (+ve) =1)

Exploratory data analysis was performed to explain each patient’s unique profile in order to better grasp the model’s specification and show the pattern of CD4 cell progression measurements taken throughout time on the patient. The graph (Fig. 1) shows the differences in CD4 cell measures of HIV/AIDS patients (both within and across patients).

### Selection and comparison of longitudinal modeling and covariance structure

The variance covariance structure model’s AIC and BIC (Additional file 1: Table S1) suggest that the association between variation in CD4 cells across time should be consistent with unstructured variance-covariance (UN) (AIC=6320). This indicates that UN did a superior job in this investigation of model explanation. Therefore, even while a patient's change in CD4 cells was connected with his or her prior CD4 cells, the association was strongest for the patient’s most recent CD4 cells and weakened as the time between counts increased (Additional file 1: Table S2).

The third model (RI-RS), in the author’s opinion, is the most frugal one for the provided dataset. As the author compares it to the other two provided random effect models, it has the smallest values of AIC (6369.8), shortest residual error (2.44), and a significant fit for change of CD4 cells (Additional file 1: Table S1).

### Predictors of CD4 cell count changes over time

For the particular change in CD4 cells of HIV/AIDS patients, the multivariable analysis of random intercept and slope (RI-RS) model is sufficient.

In order to analyze the LMM, acquire parameter estimates, and determine the model’s statistical significance predictions, the author employed the logarithmic transformation for CD4 cells (Table 3). A multivariable random intercept and slope linear mixed regression analysis regarded predictor variables to be statistically significant if their *p*-value was less than 0.05. According to the results of the multi-variable analysis, the patients’ baseline CD4 cell count and functional status of bedridden were both highly significant predictors of the change in CD4 cells over time.

**Table 3:** Parameter estimates, standard error and *p*-value for RI-RS of LMM

Fixed effect for √(CD4 cell ) count					
Parameter	Estimate	Standard Error	DF	t-value	*p-*value
Intercept	12.07	1.84	1019	6.569	0.0000*
Age	-0.013	0.0204	204	-0.61	0.54
Sex (ref.= female)					
Male	0.153	0.425	204	0.36	0.719
Residence (ref.= urban)					
Rural	0.0231	0.398	204	0.058	0.954
**Baseline CD4 cell**	0.0125	0.00125	204	9.985	0.001*
Baseline hemoglobin	0.054	0.056	204	0.98	0.32
**Function status (ref.= working)**					
**Ambulatory**	-0.952	1.0217	204	-0.9318	0.352
**Bedridden**	-2.459	1.055	204	-2.33	0.02*
WHO stage (ref.= stage-I)					
Stage-II	0.174	1.0562	204	0.165	0.869
Stage-III	0.0478	1.062	204	0.0449	0.964
Stage-IV	0.1835	1.114	204	0.164	0.869
HIV/TB status (ref.= no)					
Yes	-1.015	0.709	204	-1.43	0.154
Visiting time	0.0262	0.068	1019	0.3855	0.6999

^*^Indicates significance of covariates at 5% level of significance

A patient’s baseline CD4 cell count significantly predicts his or her recent change in CD4 cells at the 5% significance level (β = 0.0125, *P*-value = 0.001*). This suggested that there is a strong positive association between change in CD4 cell progression and baseline CD4 cell count.

Being bedridden is also shown to be a statistically significant predictor of patients changes on CD4 cells (β = -2.459, *p* = 0.02*) at the 5% significance level. They had significantly fewer CD4 cells when compared to their working functional status. While there were no differentials in age, sex, residence, baseline hemoglobin, WHO stage, HIV/TB status, or visiting time in the study (Table 3).

## Discussion

To ascertain the main predictor factors change in CD4 count, several random effects of linear mixed models were fitted in the current study. The RI-RS count model and UN variance-covariance were used in this work to fit the best correlation structure of subsequent CD4 cell assays within the same patients. This is due to the possibility of biased estimations of the model parameters caused by incorrect covariance structure specification for successive repeating measures in longitudinal analysis. The structure with the lowest Akaike information criterion (AIC) value is therefore the most preferred structure given a collection of possible covariance structures and a random effect model of LMM for the provided data set.

A statistically significant predictor of the patient’s change in CD4 cells was the baseline CD4 cell count. A better level of recovery should have been the outcome of a higher baseline CD4 cell count. This finding is consistent with those of studies carried out [13, 26].

Only bedridden patients who are HIV/AIDS patients experience a statistically significant difference in CD4 cell counts as compared to those who are functionally active. Numerous investigations [27, 28] supported the statistically significant lower predicted change of CD4 cells for bedridden functional status.

Age was found to be important predictors of CD4 count change in a cross-sectional research conducted in Ethiopia [10]. However, there was no discernible correlation between age and CD4 count change in this investigation. The huge sample size employed in this study and the discrepancy in study design could be the cause of this disagreement.

A research conducted in North Ethiopia [12] found that females responded better to ART in terms of CD4 cell counts than males, based on the demographic characteristic sex variable. This finding contradicted other studies [29, 30].

## Conclusions

In general, baseline CD4 cell and bedridden functional status were the main predictors of change in CD4 cells in the current study. Because their CD4 cell counts were lower at baseline and they had a functional status of bedridden, the majority of HIV/AIDS patients on ART had substantial predictors on the change of CD4 cell count over time.

So, public health service providers should give exceptional guidance and attention is also necessary for those patients who have lower baseline CD4 cell count and bedridden functional status.

## Limitations

The declining CD4 cell count in this study may be related to time-to-event data like survival time or time to lost follow-up following ART, which could have an impact on the findings.

## Supplementary Information

Supplementary Material 1.

## Data Availability

The data will be obtainable based on request from corresponding author of the study.

## Abbreviations

AIDS: Acquired Immunodeficiency Syndrome
HIV: Human Immunodeficiency Virus
WHO: World Health Organization
ART: Antiretroviral Therapy
SD: Standard Deviation
AIC: Akaki Information Criteria
BIC: Bayesian Information Criteria
CD4: Cluster of Differentiation Four
TB: Tuberculosis
SPSS: Statistical Software for Social Sciences’
LMM: Linear Mixed Model; RI; Random Intercept
RS: Random Slope
RI-RS: Random Intercept & Random Slope
UN: Unstructured
CSH: Heterogeneous Compound Symmetry
CS: Compound Symmetry
AR(1): First Order Autoregressive
IND: Independent
UNAIDS: United Nation report on Acquired Immune Deficiency Syndrome
