# Correction: Predictors of change in CD4 cell count over time for HIV/AIDS patients on ART follow-up in northern Ethiopia: a retrospective longitudinal study

**DOI:** 10.1186/s12865-024-00671-7

**Published:** 2024-11-25

**Authors:** Gebru Gebremeskel Gebrerufael, Zeytu Gashaw Asfaw

**Affiliations:** 1 Department of Statistics, College of Natural Science, Adigrat University, Adigrat, Ethiopia; 2 Department of Epidemiology and Biostatistics, School of Public Health, Addis Ababa University, Addis Ababa, Ethiopia

**BMC Immunology (2024) 25:64**

10.1186/s12865-024-00659-3

The original publication of this article [1] contained an incorrect affiliation for Zeytu Gashaw Asfaw. The incorrect and correct information is listed in this correction article; the original article has been updated.

Incorrect

─Zeytu Gashaw Asfaw

• 2 Department of Statistics, College of Natural and Computational Science, Hawassa University, Hawassa, Ethiopia

Correct

─Zeytu Gashaw Asfaw

• 2 Department of Epidemiology and Biostatistics, School of Public Health, Addis Ababa University, Addis Ababa, Ethiopia
