# Supplementary material for: Predictors of change in CD4 cell count over time for HIV/AIDS patients on ART follow-up in northern Ethiopia: a retrospective longitudinal study
Source: BMC Immunol. 2024 Oct 4;25:64. doi: 10.1186/s12865-024-00659-3 (PMC11451158; doi:10.1186/s12865-024-00659-3)
Supplement: Supplementary file 1 — Supplementary Material 1. [file 12865_2024_659_MOESM1_ESM.docx]

**Supplementary information**

Additional file 1: Table S1. Selection and comparison of covariance structure and LMM (RI, RS & RI-RS)

| **Linear Mixed Effect Model** (**LMM**) | | | **Information Criteria** | | | **CSH** | **UN** | **CS** | **AR(1)** | **IND** |
| --- | --- | --- | --- | --- | --- | --- | --- | --- | --- | --- |
| **Random effect** |  | **Estimates** | **AIC** | **BIC** | **AIC** | 6532 | 6320* | 6530 | 6457 | 7183 |
|  |  | **SD**  2.44  2.81  0.647  2.91  3.56  0.768  2.44* |  |  |  |  |  |  |  |  |
| RI | Intercept |  | 6451.4 | 6528.2 | **BIC** | 6598 | 6458* | 6571 | 6498 | 7219 |
|  | Residual |  |  |  |  |  |  |  |  |  |
| RS | Time |  | 6526.1 | 6602.9 |  |  |  |  |  |  |
|  | Residual |  |  |  |  |  |  |  |  |  |
| RI-RS | Intercept |  | 6369.8* | 6456.8* |  |  |  |  |  |  |
|  | Time |  |  |  |  |  |  |  |  |  |
|  | Residual |  |  |  |  |  |  |  |  |  |
| *Smallest value; RI; Random Intercept; RS: Random Slope; RI-RS: Random Intercept & Random Slope ; UN: Unstructured ;CSH: Heterogeneous compound symmetry; CS: compound symmetry; AR(1): First Order Autoregressive; IND: Independent; SD: Standard Deviation | | | | | | | | | | |

Additional file 1: Table S2. Correlation matrix on longitudinal measure on CD4 cell counts

| **Time on ART** | **Month-0** | **Month-6** | **Month-12** | **Month-18** | **Month-24** | **Month-36** |
| --- | --- | --- | --- | --- | --- | --- |
| Month-0 | 1 | 0.879 | 0.844 | 0.748 | 0.499 | 0.465 |
| Month-6 |  | 1 | 0.917 | 0.783 | 0.538 | 0.52 |
| Month-12 |  |  | 1 | 0.847 | 0.548 | 0.483 |
| Month-18 |  |  |  | 1 | 0.59 | 0.506 |
| Month-24 |  |  |  |  | 1 | 0.529 |
| Month-30 |  |  |  |  |  | 1 |
